# Preparing Medical Students for the Transition to Residency: A Qualitative Study of New Pediatric Interns

**DOI:** 10.1007/s40670-026-02703-w

**Published:** 2026-03-23

**Authors:** Nicole Meyers, Matthew Rustici, Brooke Spector, Erika Abramson

**Affiliations:** 1 Department of Pediatrics, NewYork-Presbyterian/Columbia University Irving Medical Center, 622 W 168th Street, New York, NY 10032 USA; 2 Department of Pediatrics, Northwell Health/Cohen Children’s Medical Center and Zucker School of Medicine at Hofstra University, 270-05 76th Avenue, Queens, NY United States; 3 Department of Pediatrics, NewYork-Presbyterian/Weill Cornell Medical College, 400 E 67th Street, New York, NY 10065 USA; 4 Department of Pediatrics, University of Colorado/Anschutz Medical Campus, 13001 E 17th Place, Aurora, CO 80045 USA

**Keywords:** Undergraduate medical education, Transition to residency, Graduate medical education, Qualitative research

## Abstract

**Background:**

The medical school to residency transition remains challenging for learners and educators. Within pediatrics, the effectiveness of medical school activities intended to ease this transition remains unclear, and little is known about the intern perspective. We sought to (1) describe new pediatric interns’ perspectives on areas they felt least and most prepared for and (2) elucidate the educational interventions interns found valuable during the transition.

**Methods:**

From 2/2024 to 4/2025, we conducted a qualitative study consisting of 1:1 semi-structured interviews with pediatric interns from an academic, tertiary-care medical center guided by a phenomenological approach. We utilized self-determination theory to develop our interview guide and reflexive thematic analysis to analyze transcripts.

**Results:**

Sixteen interviews were conducted with interns from 14 unique medical schools. Four themes were identified: (1) Interns felt most comfortable with medical knowledge and patient presentations, yet struggled with autonomy and counseling families in the outpatient setting; (2) Undergraduate Medical Education (UME) experiences e.g., sub-internships and Transition To Residency (TTR) courses that were pediatric specific were most valuable; (3) Early Graduate Medical Education (GME) programming should prioritize teaching institution-specific skills e.g., Electronic Medical Record (EMR) best practices; (4) A supportive environment with close peer mentoring best fosters intern growth through clinical learning. Themes informed a novel conceptual model with actionable recommendations for medical school and residency educators.

**Conclusions:**

By interviewing interns who graduated from multiple medical schools, we benefited from learners with diverse experiences. Our model should guide educators in facilitating a smoother transition to pediatric residency through programming aligned with intern needs.

## Background

The UME to GME transition remains a difficult and complex step along the learner continuum [1]. Program directors (PDs) and other educators have voiced their perspectives around students’ lack of preparedness for residency, as well as specific gaps in new interns’ knowledge and skills [2–5]. Learners have similarly shared a lack of readiness to successfully navigate the initial stages of residency [6, 7]. In fact, the Coalition for Physician Accountability formed a UME-GME Review Committee, whose 2020 recommendations include specialty-specific training in the final year of medical school to better prepare incoming interns [8]. 

There are several existing educational activities, such as TTR courses, bootcamps, and residency orientations, intended to ease this UME to GME transition and ensure students are prepared for the increase in autonomy upon starting residency. Current literature has begun to explore learner perspectives on these educational activities [6, 9, 10], which is necessary given that they are key stakeholders and must value the experiences. However, there are few studies investigating pediatric-specific experiences [11, 12], which are essential given the unique skillset pediatric interns must possess, such as proficiency in certain procedures, caring for children of different ages, and providing appropriate counseling to families [13]. 

Within pediatrics, the effectiveness of specialty-specific bootcamps designed to ease the transition to residency remains unclear. Some studies have demonstrated immediate improvements in students’ clinical skills and confidence upon completion of pediatric-specific courses [14]. However, these preparatory experiences have not yet been shown to lead to sustained knowledge gain or improved intern competence [15]. Despite this, there continues to be an expansion of TTR courses nationally [16], though with a dearth of studies exploring the pediatric intern perspective on these activities and many courses providing little pediatric-specific teaching. To further investigate the optimal combination of medical school and residency experiences to facilitate the transition from medical school to pediatric residency, we utilized qualitative methodology to elucidate the content, structure, and types of educational interventions that new pediatric interns found or would have found valuable in their transition to residency.

## Methods

### Study Design

We conducted a qualitative study with semi-structured, 1-on-1 virtual video interviews using *Zoom* (Version 6.3.11). The Weill Cornell Medicine (WCM) Institutional Review Board (IRB) approved this study as exempt (IRB 23-11026769).

### Setting and Participant Sampling

Study participants were categorical pediatric residents in their first year of training (interns) from the 2023–2024 and 2024–2025 classes at WCM, which is an academic, tertiary-care medical center in New York City. We utilized criterion sampling to recruit interns who were at least 2 months into their intern year to ensure they had sufficient exposure to thoughtfully comment on their preparedness for residency. We included interns who attended medical school in the United States, including at WCM. We recruited via e-mails from chief residents and a study team member (N.M.), who was intentionally chosen given she was not a faculty member or program leader. Participation was voluntary and interns received a $25 gift card as an incentive for participation. Recruitment materials emphasized that program leaders and faculty would not be aware if any specific intern did or did not participate in the study and thus participation would have no bearing on any future evaluation.

### Interview Procedure

From February 2024 to April 2025, a single study investigator (N.M.) who had prior experience as a qualitative interviewer obtained oral consent from participants and performed all *Zoom* interviews. We used a phenomenological approach to better understand the pediatric intern perspective on the activities that eased their transition to residency. The semi-structured interview guide (available as Appendix 1) was informed by self-determination theory (SDT), which is a leading theory on human motivation and wellness, and commonly utilized within health professions education. SDT argues that optimal intrinsic motivation requires support in three domains: competence, autonomy, and relatedness. It highlights the importance of autonomy-supportive teaching, as well as the need to fully understand what learners want and need from their education, thus aligning well with our study objectives [17–19]. Our interview questions centered around how medical school and early residency experiences foster competence, autonomy, and relatedness among students as they transition to pediatric interns. Our guide underwent iterative review by the Vice Chair of Health Services and Medical Education Research and Associate Dean of Curricular Affairs for Weill Cornell Medical College (E.A.), as well as a TTR course director and national expert in pediatric bootcamps (M.R.). The questions were piloted with a pediatric chief resident and revised to ensure clarity and understandability.

### Reflexivity

We recognized the potential power dynamic between senior faculty (E.A. and B.S.) and interns from the same institution, thus only N.M. conducted interviews. We maintained confidentiality by assigning each transcript a unique study code and removing all potentially identifying information. No names were collected during the interview and the only potentially identifying information collected –– medical school location and size –– was used solely by N.M. to determine the number of distinct medical schools represented and was removed from the final interview transcript reviewed by other study investigators. Of note, two coding team members (B.S. and M.R.) spend significant time designing and implementing TTR curricula as course directors at their respective institutions, which provided a valuable perspective on our results. Our coding team engaged in frequent discussions to foster reflexivity around how their roles and any associated beliefs may bias our analyses. All transcripts were initially coded independently and then discussed as a group to ensure a single study team member’s perspective was not overly represented.

### Data Analysis

Audio recordings were transcribed using the *Zoom* transcription feature and then manually reviewed by N.M. for accuracy. We analyzed interview transcripts using reflective thematic analysis informed by Braun and Clarke’s six-step approach [20]. All study investigators (N.M., M.R., B.S. and E.A.) reviewed early transcripts for frequently seen phrases and concepts to generate an initial codebook. These codes were generated inductively from the data itself, as well as deductively from the initial SDT framework to ensure completeness. The codebook underwent iterative review and was applied to all data. *Dedoose* [21] was used to compile all transcripts, codes and quotations. All investigators then identified patterns within the codes to generate broader themes, which were continuously refined based on frequent discussions among team members and re-review of the transcripts themselves. Interns were recruited until thematic saturation was achieved. Based on these themes, we constructed a novel conceptual model for educators developing UME and early GME curricula aimed at easing interns’ transition to pediatric residency. N.M. shared the themes and conceptual model with several interviewees as a post-hoc member-checking exercise to confirm the trustworthiness of our findings.

## Results

Sixteen semi-structured interviews were conducted among interns from the 2023–2024 and 2024–2025 classes (*N  = 41)* with a mean length of 25–26 min. Interns graduated from 14 distinct medical schools and were on average 7 months into their intern years. 81% (13/16) of interns reported participation in TTR courses, which they described as highly variable regarding pediatric-specific content, pedagogy, time of year, and duration. Interns also described unique experiences during pediatric sub-internships (sub-Is) as far as degree of autonomy, specific responsibilities, and patient volume.

Four major themes were identified: (1) Interns felt most comfortable with their medical knowledge and oral patient presentations, while they struggled acting as autonomous providers and counseling families in the outpatient setting; (2) UME experiences, such as sub-Is and TTR courses, emphasizing pediatric-specific topics were most valuable in easing the TTR and fostering professional identity formation; (3) Early GME programming should prioritize distinct topics from that of UME, such as institution-specific skills, in order to best prepare interns for their new role; (4) A supportive environment with close peer mentoring best fosters intern growth through clinical learning. Table 1 describes these themes, as well as sub-themes and representative quotes.

**Table 1:** Qualitative themes, sub-themes and representative quotes

Theme	Sub-Theme	Representative Quote
Interns felt comfortable with their medical knowledge and oral patient presentations, while they struggled acting as autonomous providers and counseling families in the outpatient setting	Comfortable developing differentials/plans and presenting patients	“What felt a little more like a next step was for me to start coming up with differentials and plans - that seemed less stressful than all of the Epic chats.” (P3)
	Struggled with acting autonomously and performing activities of daily interning	“The most challenging thing was learning to trust my gut and realizing that I am the doctor, I am the first-line physician taking care of my patients so trying to present my ideas less as questions and more as suggestions.” (P13) “The difficult parts of being an intern were the little nuances of learning how to put in an order or where this thing is in the hospital or learning what your exact roles are as an intern.” (P11)
	Found outpatient setting to be challenging	“What I was most unprepared for was the outpatient setting and not having much exposure to that and not knowing a lot of the answers to parenting questions.” (P10)
UME experiences, such as sub-Is and TTR courses, emphasizing pediatric-specific topics were most valuable in easing the TTR and fostering professional identity formation	Pediatric sub-Is	“As a sub-I, I was treated like a resident with a lower patient load so I felt pretty well-prepared to know exactly what tasks I needed to do.” (P10)
	Pediatric-specific simulations during TTR courses	“Going through the sims was helpful to start having that experience with what to do when you are in the room with the patient and something is going wrong or the patient is not getting better. Also being with my peers and talking about when to ask for help, who to ask for help like things that we should start to get comfortable with like asthma and seizures.” (P8)
	Professional identity formation via TTR courses	“We tried tiny samples of liquid antibiotics - like those things that are very unique to pediatrics where you are going to have to give your 3-year-old patient something that tastes horrible and now you know what it tastes like - that made me really excited to start intern year.” (P15) “I was really excited to know that I'd be working with people [classmates going into pediatrics] just as interested and excited to work with and treat kids.” (P9)
Early GME programming should prioritize distinct topics from that of UME, such as institution-specific skills, in order to best prepare interns for their new role	EMR best practices	“During orientation, we probably had a lecture on Epic but if there's a computer lab, a session where we're on the computer and doing it actively with an Epic person would be interesting like a simulation of Epic, that would be more useful than just looking at slides of where to find things.” (P14)
	Rotation-specific logistics	“Something that I think could be helpful is having pre-briefing before rotations - if you sat down with a co-resident or a fellow and you were like you're starting in the emergency room, what should you expect? What will your days be like?” (P8)
	Personal challenges and available mental health resources	“In orientation - we learned about the [mental health program] that [institution] has and having either fellows or attendings or even co-residents talk to the incoming interns that these are some of the personal challenges that people face - how do people cope, what are the resources there I think could be helpful.” (P8)
A supportive environment with close peer mentoring best fosters intern growth through clinical learning	Increased confidence via repeated practice during intern year	“There's so much knowledge gained within that 1 year and so that was really impressive to me that my second years know so much and now I'm realizing I know so much more than I did 6 months ago.” (P10)
	Value of a supportive environment and culture of teamwork	“For a few of my co-residents who were on the floors - attendings would write their notes if it was their first week of their first block allowing them to focus on how to do tasks, how to respond to messages, or their seniors would have them forward all of their messages - these are very niche small ways to feel support.” (P15)
	Importance of mindset e.g., openness to learn as some skills cannot be taught in advance	“In terms of fund of knowledge - I feel like those are things you just have to jump in to learn and get your feet wet.” (P14)

### Theme 1. Interns Felt Comfortable With Their Medical Knowledge and Oral Patient Presentations, While They Struggled Acting as Autonomous Providers and Counseling Families in the Outpatient Setting

Interns reported comfort around skills they had ample prior experience with, such as oral patient presentations, developing differentials and plans, and accessing resources to answer clinical questions. One described “I felt pretty prepared for patient presentations. That was something that was both in my clinical clerkships and practice-based learning - a small group session 2 times a week in med school.” (P13) Another reported “The most challenging things were less so the medical knowledge but more so the resident-oriented tasks. As for applying the clinical knowledge - I was more familiar with how to have resources to look up that information.” (P1).

Interns described their newfound autonomy as particularly challenging given it was unique from the medical student role. One reported “It was a struggle trying to be that first one to answer questions while you’re in the patient’s room because I think as a med student, I could get to a certain point and lean on my residents, but then as the intern, that became my responsibility to answer questions.” (P2) Interns also struggled with typical resident tasks, which included order-writing, discharge logistics e.g., acquiring medications/supplies, and care coordination.

Many residents reported limited exposure to the outpatient setting during medical school and the provision of age-appropriate anticipatory guidance and counseling as an intern to be especially difficult. One described: “I found primary care visits in the beginning to be challenging. There’s a lot of counseling that goes into primary care visits and that transition where you are suddenly the PCP - I had more minimal experience with that so it was something new that I found challenging.” (P7) and another said “Going into clinic, I was pretty unprepared for how to properly advise parents and when they were asking if this was normal or not.” (P9).

### Theme 2. UME Experiences, Such as Sub-Is and TTR Courses, Emphasizing Pediatric-specific Topics Were Most Valuable in Easing the TTR and Fostering Professional Identity Formation

Interviewees almost universally agreed on the benefit of pediatric sub-Is in easing their transition to residency. “One of the best things was doing my sub-I. The responsibilities that they gave me for my patients when it came to pre-rounding, rounding and checking in on them in the afternoons - I felt like an intern at that time so I felt pretty prepared for that.” (P5) They emphasized that the autonomy and experiences granted on their sub-Is with calling consults and pending orders, translated to increased comfort with these resident-specific tasks during intern year.

Interns valued pediatric-specific simulations during their TTR courses citing how they were realistic, relevant, and offered opportunities to practice their escalation and teamwork skills. One described their TTR course: “Most of it was focused on simulations, so I think it was really helpful for the worst-case scenario and feeling a lot more comfortable and confident with contingency planning for some of the common things like asthma and anaphylaxis.” (P12) To the contrary, a different participant emphasized the limited value of specialty-agnostic TTR courses: “We spent the majority of the time talking about things that were unrelated to pediatrics. We were going over common dosages and it was all adult dosages. I might as well not be at the session because none of that is going to matter to me.” (P2).

Many participants shared how pediatric-specific TTR courses fostered professional identity formation through re-immersion into the field of pediatrics alongside future colleagues. One shared: “I hadn’t been in the pediatric realm in quite a few months – it was really nice to think about pediatric patients, specifically getting the history from multiple family members near the mannequin.” (P9) and another explained that the most exciting part of their TTR course was “the energy of these are my future colleagues and I’ll probably see them at conferences - I think just being in a space where I was with all my classmates and we were all going into a similar career journey.” (P14).

### Theme 3. Early GME Programming Should Prioritize Distinct Topics From that of UME, Such as Institution-specific Skills, in Order to Best Prepare Interns for Their New Role

Participants emphasized that some elements are better taught and more relevant in the GME rather than UME setting. Interns recommended that residency orientations emphasize institution-specific skills, such as best practices around electronic medical record (EMR) use, including admission workflows and frequently-used note templates. One intern shared that EMR training “would have been most helpful for the residency program to do in the beginning because at the end of medical school – training on one EMR and sending people to different ones may not be very helpful.” (P12) They suggested near-peer modeling of how to approach day-to-day logistics on different rotations, especially when it was an intern’s first time on a particular service. One describes “It would have been helpful at the beginning of residency to learn some tricks instead of showing up to each rotation and being like how do you do all these orders? How do you do all your notes?” (P12).

Interns also mentioned the importance of “the personal transition of basically not working at all to now working 6 days a week and you don’t get all your weekends off. Everyone theoretically knows that going in and I was expecting that, but some people might find it harder than they expected and so just always making sure that people feel supported around that time, not just in their clinical sense, but how they personally are doing is nice.” (P11) They emphasized the importance of promoting awareness around institution-specific mental health resources, while recognizing that it is difficult to appreciate personal struggles until actually experiencing them.

### Theme 4. A Supportive Environment With Close Peer Mentoring Best Fosters Intern Growth Through Clinical Learning

Participants shared how experiences in their new intern role were sometimes more helpful for their professional growth than teaching during medical school or residency orientation. Interns underscored the importance of a supportive, safe learning environment and culture of teamwork in building confidence while in this new role. “I think the support I got as I was transitioning made it so much easier. Every day I have a better idea of what I’m doing and what to expect. The big scary thing was the unknown - not even knowing what a day in the ED looked like and then now even after my first week, understanding what I should expect going in and the workflow of seeing somebody, presenting to your attending, working with them, writing notes, filling in the gaps of everyone working together as a team.” (P11) Another intern emphasized the importance of supportive peer relationships by saying “If there’s something going on in life or going on in the hospital that you feel underprepared to handle - you can go to your senior, you can go to somebody else to support you - that is a big component of the transition into intern year.” (P15).

Interns also shared the importance of values, e.g., an openness and eagerness to learn, as there are some skills that simply can’t be taught in advance. “The best way to transition is honestly just to jump off the boat because that’s the best way to really learn. We can talk about it and look at lectures and ask people their experiences, but you’re never really going to learn until you do it yourself.” (P14).

### Model for UME and GME Educators to Support the Transition to Pediatric Residency

**Fig. 1:**
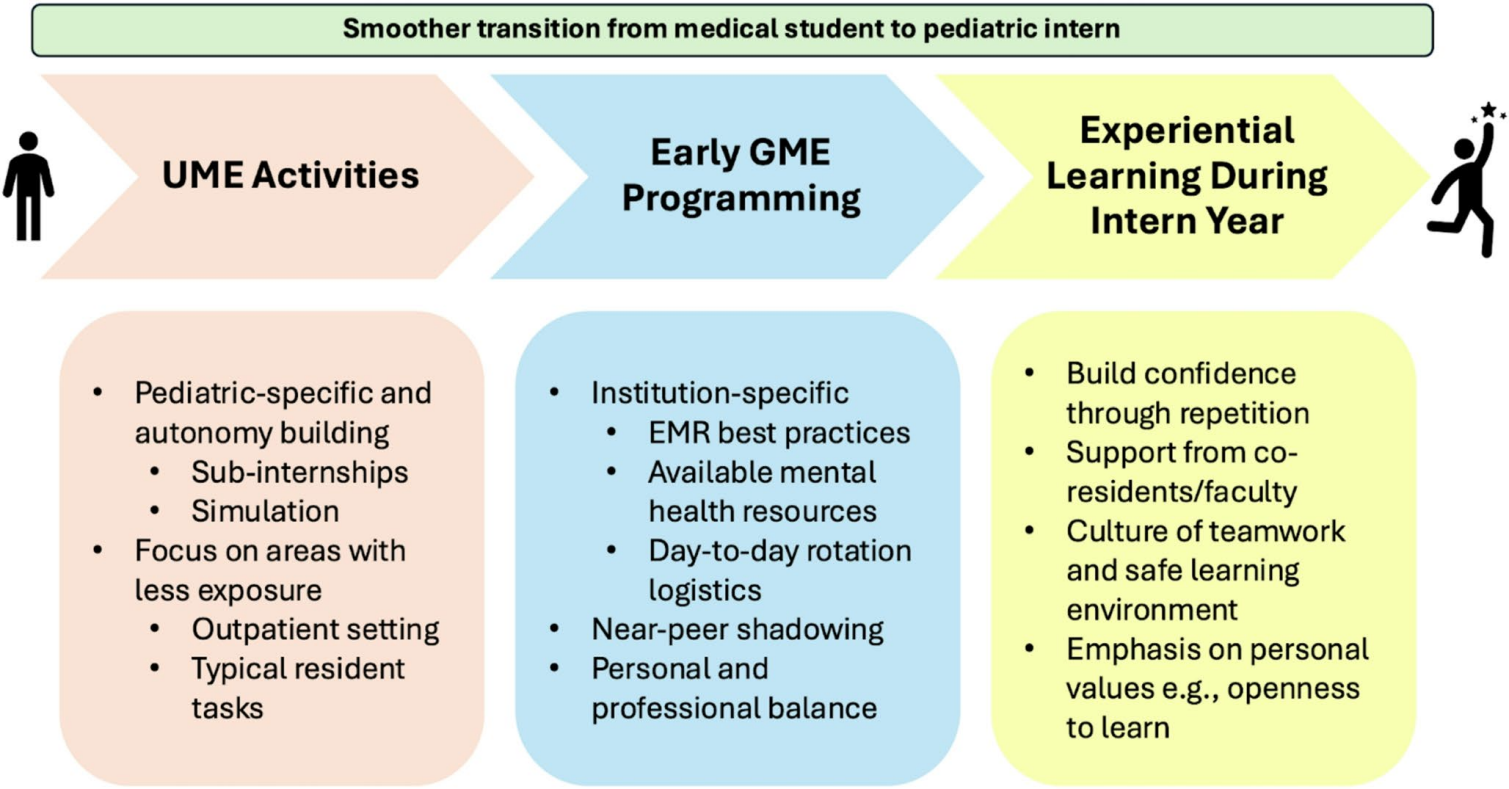
Model for UME and GME educators to support the transition to pediatric residency

With these themes in mind, we developed a novel conceptual model for how UME and GME initiatives can work on a continuum to support the transition to pediatric residency (Fig. 1). Medical school educators should focus on encouraging autonomy and professional identity formation through pediatric-specific content via sub-internships and TTR courses. They should thoughtfully incorporate greater exposure to areas students have less experience with, such as the pediatric outpatient setting and typical resident activities, e.g., order-writing and discharge preparation. In the next stage of early GME programming, interns seek institution-specific teaching to better integrate them into an often brand-new system. Residency educators must be aware of these challenging areas for new interns, as well as the importance of fostering a team-oriented culture and safe learning environment during this time of significant growth. Enhanced UME TTR curricula, thoughtful early GME programming, and a supportive residency infrastructure must work in concert to successfully ease interns’ transition to pediatric residency.

## Discussion

Pediatric interns from a multitude of medical schools shared their experiences around the transition to residency, specifically the areas they felt most prepared for (oral patient presentations) and least prepared for (the outpatient setting). They described the pediatric-specific UME experiences valuable in easing their transition, as well as the GME programming and supportive culture necessary to foster their growth. These perspectives informed the development of a model conceptualizing how UME and GME efforts can ease this challenging transition, which includes specific recommendations for educators in this space.

A recent Delphi consensus study surveying TTR course directors and residency PDs established a set of skills to guide general TTR course development, many of which mirrored those our pediatric interns felt unprepared for, including discharge logistics, personal wellness, and effective use of the EMR [22]. Our interviewees also endorsed challenges in other areas, including daily intern tasks, such as order-writing, and counseling in the outpatient setting, which may represent unique insight from the pediatric intern perspective.

Another recent publication compiled recommendations from several TTR course directors, and emphasized the need for TTR courses to be specialty-specific with opportunities for active learning [23]. This closely aligns with SDT’s focus around autonomy and competence [19], and our participants’ perspectives around the value of autonomy-building and pediatric-specific UME experiences in encouraging professional identity formation. Our intern cohort repeatedly shared the benefit of TTR courses with a pediatric emphasis, in contrast with the limited utility of TTR courses that were specialty-agnostic. Despite the perceived value of sub-Is and pediatric-specific TTR courses among our interviewees, one study found few educationally significant associations between participation in these activities and intern performance [15]. Rideout et al. compared intern performance via faculty assessment of specific milestone-based sub-competencies after interns’ first inpatient rotation between residents who did or did not participate in a pediatric TTR course. The discrepancy between our study findings and that of Rideout et al. may be due to differing outcome metrics, given they assessed competence versus our study assessed resident perceptions of their own skills. Their lack of positive findings may also underscore the need to continue improving these activities using learner perspectives including those gained from our intern cohort.

In terms of recommendations for GME educators, our study suggests that several of the topics mentioned in the Rustici et al. study above, such as personal well-being, day-to-day rotation logistics, and EMR strategies, may be better integrated into early residency programming to ensure relevance at interns’ respective institutions. The Council on Medical Student Education in Pediatrics recommends that UME bootcamps emphasize topics such as wellness and EMR use [24], however our study suggests these may be better suited for GME programming. In fact, the Alliance for Academic Internal Medicine published a framework based on expert opinion for GME orientation curricula in their field, highlighting the need to focus on personal well-being and EHR specifics [25]. 

Our model’s emphasis on the importance of a supportive environment during this time of rapid professional development aligns with existing literature on the value of coaching in fostering individualized, self-reflective growth during the TTR [26]. The usefulness of near-peer advice and team-building, which aligns with SDT’s need for relatedness [19], emerged in prior qualitative studies of trainees from diverse specialties [27, 28], though the potential value of resident shadowing may be novel among our pediatric-specific cohort. The importance of personal values such as openness to learning, which was identified by our interviewees as critical to easing the transition, seems to be explored less in current TTR literature. Ensuring this growth mindset is a cornerstone of successful coaching [26] and thereby further supports the value of this approach in the TTR.

By interviewing new interns who graduated from multiple medical schools, we benefited from learners with an array of experiences and unique perspectives on the TTR. However, our study is not without limitations. Pediatric interns were recruited from a single program, which may limit generalizability to those from institutions less like ours. All interviews were conducted by a single interviewer (N.M.), which ensured consistency, though it may have introduced repeated bias. N.M. was intentionally chosen as the sole interviewer to minimize any potential power dynamic as she was the only non-faculty study team member. Our group engaged in a reflexivity exercise to mitigate biases throughout both the data collection and analysis phases. Demographics, aside from location and size of medical school attended, were not collected to maintain anonymity in a relatively small group of subjects. Given the nature of semi-structured interviews, we were unable to quantify complete information about TTR and sub-I exposure.

## Conclusions

We build on existing literature by developing a model coordinating UME and GME efforts to support the transition to pediatric residency. We provide actionable recommendations for pediatric educators to ease this challenging transition through a multi-faceted approach informed by trainees’ perspectives. Our insights should guide the development, standardization, and enhancement of UME and GME curricula around the transition to pediatric residency. In fact, several of our findings are applicable to specialties beyond pediatrics and could inform broader TTR curriculum reform. As these educational activities undergo iterative review, it will be imperative to continue investigating their perceived value and efficacy by learners and educators alike.

## Appendix 1. Semi-structured Interview Guide

The goal of this interview is to think about your transition from being a medical student to an intern. Specifically, we are looking to better understand the content, structure, and types of medical school curricula that you found most valuable in your transition to residency.

- We will start with a few questions about the medical school you attended. What was the approximate size of your class? Where was it located?
- Tell me about your transition from medical school to the first few months of residency.
  - What was most challenging around the new responsibilities?
  - What did you feel least prepared for?
  - What did you feel most prepared for?
  - Was there anything that surprised you during this transition that you weren’t expecting?
- Many medical schools have specific courses and experiences designed to help prepare students for the transition to residency. What did your medical school offer?
  - What was most effective and why?
  - What was least effective and why?
  - What, if any aspects, of the curricula do you feel best prepared you to assume the increased responsibilities of an intern?
  - What, if any aspects, of the curricula made you most excited to be a pediatrician?
  - If you were responsible for curricula at your institution designed to prepare students to become pediatric residents, what would it look like?
- What other elements of the transition from medical student to intern do you feel are important that we haven’t yet discussed?
